# Antimycobacterial and Anti-inflammatory Mechanisms of Baicalin via Induced Autophagy in Macrophages Infected with *Mycobacterium tuberculosis*

**DOI:** 10.3389/fmicb.2017.02142

**Published:** 2017-11-02

**Authors:** Qingwen Zhang, Jinxia Sun, Yuli Wang, Weigang He, Lixin Wang, Yuejuan Zheng, Jing Wu, Ying Zhang, Xin Jiang

**Affiliations:** ^1^Department of Immunology and Microbiology, School of Basic Medical Sciences, Shanghai University of Traditional Chinese Medicine, Shanghai, China; ^2^Department of Infectious Diseases, Institute of Infectious Diseases, Huashan Hospital, Fudan University, Shanghai, China; ^3^Department of Molecular Microbiology and Immunology, Bloomberg School of Public Health, Johns Hopkins University, Baltimore, MD, United States

**Keywords:** baicalin, *Mycobacterium tuberculosis*, host-directed therapy, autophagy, inflammasome

## Abstract

Tuberculosis (TB) remains a leading killer worldwide among infectious diseases and the effective control of TB is still challenging. Autophagy is an intracellular self-digestion process which has been increasingly recognized as a major host immune defense mechanism against intracellular microorganisms like *Mycobacterium tuberculosis* (Mtb) and serves as a key negative regulator of inflammation. Clinically, chronic inflammation surrounding Mtb can persist for decades leading to lung injury that can remain even after successful treatment. Adjunct host-directed therapy (HDT) based on both antimycobacterial and anti-inflammatory interventions could be exploited to improve treatment efficacy and outcome. Autophagy occurring in the host macrophages represents a logical host target. Here, we show that herbal medicine, baicalin, could induce autophagy in macrophage cell line Raw264.7 and caused increased killing of intracellular Mtb. Further, baicalin inhibited Mtb-induced NLRP3 inflammasome activation and subsequent inflammasome-derived IL-1β. To investigate the molecular mechanisms of baicalin, the signaling pathways associated with autophagy were examined. Results indicated that baicalin decreased the levels of phosphorylated protein kinase B (p-Akt) and phosphorylated mammalian target of rapamycin (p-mTOR) at Ser473 and Ser2448, respectively, but did not alter the phosphorylation of p38, JNK, or ERK both in Raw264.7 and primary peritoneal macrophages. Moreover, baicalin exerted an obvious inhibitory effect on nuclear factor-kappa B (NF-κB) activity. Finally, immunofluorescence studies demonstrated that baicalin promoted the co-localization of inflammasome with autophagosome may serve as the underlying mechanism of autophagic degradative effect on reducing inflammasome activation. Together, baicalin definitely induces the activation of autophagy on the Mtb-infected macrophages through PI3K/Akt/mTOR pathway instead of MAPK pathway. Furthermore, baicalin inhibited the PI3K/Akt/NF-κB signal pathway, and both autophagy induction and NF-κB inhibition contribute to limiting the NLRP3 inflammasome as well as subsequent production of pro-inflammatory cytokine IL-1β. Based on these results, we conclude that baicalin is a promising antimycobacterial and anti-inflammatory agent which can be a novel candidate for the development of new adjunct drugs targeting HDT for possible improved treatment.

## Introduction

Tuberculosis (TB) continues to be a major cause of significant morbidity and mortality world-wide. The latest World Health Organization (WHO) report indicates that TB remains a global emergency (WHO, 2016). New available anti-TB drugs are limited in number and activity and they mostly direct microbial targets and have faced many obstacles (Zumla et al., 2015) such as increasing drug resistance, complex drug regimens, lengthy, and toxic treatment durations. Recent work on host immunity, host-pathogen interactions and host-directed interventions have shown that supplementation anti-TB therapy with host modulators, such as imatinib and nitazoxanide may shorten the treatment times, reduce the lung damage caused by inflammation, and lower the risk of relapse or reinfection (Hawn et al., 2013). Host-directed therapy (HDT) is a new strategy for adjuvant therapy in fighting against TB, which focuses on potentiating key components of host antimycobacterial effector mechanisms, while restricting inflammation and pathological damage in the lung (Hawn et al., 2013; Zumla and Maeurer, 2016; Machelart et al., 2017; Yang, 2017).

Autophagy is a highly conserved and fundamental biological process in eukaryotic cells (Mizushima et al., 2008). Most of autophagic physiological effects, such as maintaining cell, tissue, and organism homeostasis, are the result of its degradative activities (Boya et al., 2013), while the unconventional function of autophagy such as biogenesis and secretory roles in protein processing are beginning to be recognized (Deretic et al., 2012; Boya et al., 2013). A cardinal structural and functional feature of autophagy is the formation of bilayer membrane organelles called autophagosomes. The generation of LC3-II is an emblematic event associated with autophagy and the reduction of p62, a specific substrate protein of autophagosome, signifies the generation of highly lytic degradative organelles, autolysosome, resulting from the fusion of autophagosome with lysosome. Increasing evidences have shown antimicrobial role of autophagy against *Mycobacterium tuberculosis* (Mtb) (Gutierrez et al., 2004; Singh et al., 2006; Bradfute et al., 2013). The mechanism of killing of intracellular mycobacteria by autophagy is based on the strong degradative and other antimicrobial properties unique to autolysosome (Ponpuak et al., 2010). Autophagy eliminates mycobacteria through several mechanisms. First, induction of autophagy indirectly promotes maturation of Mtb phagosomes into degradative organelles (Harris et al., 2007; Fabri et al., 2011), conquering the well-known Mtb-mediated phagosome maturation arrest. Second, autophagosomes directly capture a subset of intracellular Mtb that then progress into degradative autolysosomes (Gutierrez et al., 2004; Watson et al., 2012). Third, autophagy has a bactericidal effect relying on the classical antimicrobial peptides such as cathelicidin through fusion with lysosomes where cathelicidin is stored or neo-antimicrobial peptides produced through autophagic proteolysis of innocuous cytosolic proteins such as ubiquitin (Alonso et al., 2007) and ribosomal proteins (Yuk et al., 2009; Ponpuak et al., 2010; Fabri et al., 2011). Thus, autophagy can be triggered by immunological and physiological stimuli enabling macrophages to kill intracellular Mtb.

The activation of autophagy can be regulated by a wide variety of signals (He and Klionsky, 2009; Yang and Klionsky, 2009; Yin et al., 2016). The kinase mTOR is a major modulator of autophagy and it receives inputs from different signaling pathways, and is a downstream target of the phosphatidylinositol 3 kinase (PI3K)/protein kinase B (Akt) pathway. The PI3K/Akt/mTOR signaling pathway has been recognized to negatively regulate the activation of autophagy (Heras-Sandoval et al., 2014). Moreover, the activation of mitogen activated protein kinases (MAPK) pathway can induce autophagy (Krishna and Narang, 2008; Zhou et al., 2015). MAPK is a well-known serine/threonine protein kinase, the associated signal pathway is one of the most important regulatory mechanisms in eukaryotic cells, with p38, JNK, ERK1/2 being the key members of MAPK sub-families (Krishna and Narang, 2008). Additionally, PI3K/Akt pathway also contributes to the activation of NF-κB by inducing the phosphorylation level of IKKα/β and IκBα (Kang et al., 2012; Guo et al., 2016).

Macrophages infected with Mtb secrete proinflammatory cytokines, including IL-1β and IL-18 (Koo et al., 2008; Kleinnijenhuis et al., 2009). The increase of IL-1β could trigger other immunological and inflammatory cells to synthesize proinflammatory cytokines including TNF-α, IL-6 et al, causing subsequently inflammatory and immunological damage (Dinarello, 1996; Dorhoi and Kaufmann, 2016). Although the production of IL-1 and TNF-α, as well as other proinflammatory cytokines is designed to be protective, if left unchecked, their excessive or inappropriate production may lead to severe inflammatory diseases (Beutler and Cerami, 1988; Dorhoi and Kaufmann, 2014). Relevant to Mtb infection, IL-1-coated beads are capable of inducing large granulomas in lung tissue (Kasahara et al., 1988). Furthermore, production of elevated TNF-α could cause severe inflammation in vital organs (such as lungs and spleen) and leading to early death (Bekker et al., 2000). It has been shown that Mtb activates NLRP3 inflammasome and results in the production of mature IL-1β in infected macrophages (Mishra et al., 2010; Wong and Jacobs, 2011; Dorhoi et al., 2012). The inflammasome is a macromolecular protein complex consisting of at least three components: a NLR (NOD-like receptor) protein such as NLRP3, apoptosis-associated speck-like protein containing a caspase recruitment domain (ASC), and pro-caspase-1. Upon activation by agonists, the inflammasome processes pro-IL-1β into a mature, biologically active IL-1β for secretion extracellularly (Lamkanfi and Dixit, 2014; He et al., 2016). The NLRP3 inflammasome can be regulated by NF-κB pathway where synthesis of NLRP3 and pro-IL-1β provides the first signals for inflammasome activation (Ghonime et al., 2014; Patel et al., 2017). Interestingly, a number of consistent reports have unequivocally indicated that autophagy plays a negative role in the process of inflammation by inhibiting the releasing of IL-1β (Lupfer et al., 2013; Martins et al., 2015; Saitoh and Akira, 2016). Loss of autophagy (ATG16L1 deficiency) increases IL-1β levels which aggravates the degree of inflammation in a mouse gut inflammation model (Saitoh et al., 2008). Autophagy inhibits the production of IL-1β indirectly, by lowering the endogenous stimuli of inflammasome activation (Nakahira et al., 2011; Zhou et al., 2011) and may also directly, via autophagic degradation of inflammasome components (Harris et al., 2011; Shi et al., 2012). Consistently, there are studies which have shown that autophagy protects from excessive inflammation during Mtb infection (Castillo et al., 2012) and protects against Mtb pathogenesis *in vivo* (Castillo et al., 2012; Watson et al., 2012). Thus, autophagy plays an important role in fighting against TB by direct killing the pathogen while preventing excessive inflammatory injury as well. In fact, as an adjunctive therapy, it has been demonstrated that autophagy contributes to the efficacy of frontline anti-tuberculosis chemotherapeutics, such as isoniazid and pyrazinamide (Kim et al., 2012). Thus, pharmaceutical manipulation on autophagy could be potentially useful as new strategies in anti-tuberculosis chemotherapeutics.

Baicalin is a flavonoid isolated from the extracts of dried roots of Scutellaria baicalensis Georgi (Huang Qin), a plant that belongs to the labiatae family, and its chemical structure has been verified (de Oliveira et al., 2015). Baicalin possesses many biological activities such as antibacterial, anti-inflammatory, anti-allergic, anti-spasmodic, and anti-cancer (Srinivas, 2010; Yu et al., 2013). Furthermore, baicalin could induce autophay in cancer (Zhang et al., 2012; Lin et al., 2013) causing subsequent autophagic tumor cell death. The molecular mechanisms of bacalin-induced autophagy in cancer cells involves blocking of the Akt signaling (Lin et al., 2013) and downregulation of CD147 (Zhang et al., 2012). The present study was carried out to investigate the mechanism of the immunological protective effect of baicalin in macrophages infected with Mtb.

## Materials and methods

### Mice and reagents

Female C57BL/6 J mice (4–8 weeks of age, weight 20 ± 3 g) were obtained from Vital River Laboratory Animal Technology Co., Ltd. (Beijing, China). All mice were acclimated for at least 1 week before the experiments and housed in a pathogen-free facility. Animal experiments were carried out in strict accordance with the National Institute of Health Guide for the Care and Use of Laboratory Animals, with the approval of the Scientific Investigation Board of Shanghai University of Traditional Chinese Medicine (Shanghai, China). DMSO, bovine serum albumin (BSA) and OPTI-MEM medium were purchased from Sigma (St. Louis, MO). RIPA lysis buffer, BCA Protein Assay Kit and Protein A/G agarose/sepharose beads were obtained from the Beyotime Institute of Biotechnology (Shanghai, China). The following antibodies were used: anti-NLRP3 (cat. #15101), anti-IL-1β (cat. #12507), anti-ASC (cat. #67824), anti-LC3 (cat. #2775), anti-p62 (cat. #5114), anti-Akt (cat. #4691), anti-phosphorylated Akt (Ser473) (cat. #4060), anti-mTOR (cat. #2972), anti-phosphorylated mTOR (Ser2448) (cat. #5536), anti-phosphorylated p38 (cat. #4511), anti-phosphorylated JNK (cat. #4668), and anti-phosphorylated ERK1/2 (cat. #4370) were purchased from Cell Signaling Technology, Inc. (CST, Danvers, MA, USA); rabbit anti-caspase-1 (cat. #sc-514), goat anti-rabbit (cat. #sc-2012), donkey anti-goat (cat. #sc-2094), and goat anti-mouse LC3 (cat. #sc-16755) were purchased from Santa Cruz Biotechnology, Inc. (Santa Cruz CA, USA); donkey anti-rabbit IgG H&L antibody (conjugated with Alexa Fluor® 488; ab150073) and donkey anti- goat IgG H&L antibody (conjugated with Alexa Fluor® 647; ab150131) were acquired from Abcam (Cambridge, UK); anti-β-actin (cat. #66009-1-lg) monoclonal antibody was from ProteinTech Group (Chicago, IL). Baicalin (Molecular Weight: 446.36, purity > 98%) was purchased from shanghai tauto biotech co., LTD. (shanghai, China). Dulbecco's Modified Eagle's Medium (DMEM) was obtained from HyClone Laboratories, Inc (Logan, UT, USA). Middlebrook 7H9 and 7H10 media were obtained from Difco (Detroit, MI, USA) and oleic acid-albumin-dextrose-catalase (OADC) supplements were from BD Biosciences (BD, Sparks, MD, USA).

### Cell culture

The Raw264.7 murine macrophage cell line was cultured in DMEM supplemented with 10% fetal bovine serum (FBS) in 5% CO2 at 37°C. Thioglycolate-elicited mouse primary peritoneal macrophages were prepared from female C57BL/6 J mice as described previously (Jiang et al., 2017). After 2 h, non-adherent cells were removed and the adherent cells were used as primary peritoneal macrophages.

### CCK-8 assay for cell viability

Raw264.7 (1 × 10^4^ cells/100 μl) cells were seeded into 96-well culture plates overnight at 37°C and atmospheric conditions of 5% CO_2_. The culture medium was then replaced with medium containing different concentrations of baicalin (0, 12.5, 25, 50, 100 μM) for 24, 48, 72 h. At the end of the culture, 10 μl of the CCK-8 reagent was added to each well. After 1–2 h of incubation at 37°C, the absorbance was determined at 450 nm using a Synergy 2 Microplate Reader (Bio-Tek, USA).

### Bacterial strains

The Mtb H37Ra was used in this study. H37Ra strain was grown in Middlebrook 7H9 or 7H10 broth supplemented with 0.2% glycerol, 0.05% Tween-80, and 10% Middlebrook OADC supplement.

### Mtb infection

The Raw264.7 or primary peritoneal macrophage cells were seeded at various specifications of the cell culture plates and grown at 37°C overnight. The cells (1 × 10^6^, 1 × 10^5^, or 5 × 10^5^) were infected with Mtb H37Ra (MOI = 10). After 4 h of co-incubation at 37°C, cells were washed three times with sterile phosphate-buffered saline (PBS) and cultured with DMEM containing 10% FBS in the presence and absence of different concentrations of baicalin (0, 12.5, 25, 50, or 100 μM) for different times.

### Colony forming unit (CFU)

Raw264.7 cells (5 × 10^5^) were seeded in six-well plates, after infection for 4 h (MOI = 10), cells were washed with sterile PBS and added new complete medium (10% FBS) into the plates in the presence or absence of baicalin (100 μM). After 48 h, the cells were ruptured (0.1% Triton X-100) to release intracellular bacteria and diluted to appropriate dilutions with sterile PBS for CFU count on 7H10 agar plates.

### Western blot

Cells were collected and lysed in lysis buffer, and then the whole cell lysate was separated by SDS-PAGE and further transferred onto nitrocellulose membranes. After blocking with TBST (0.5% Tween-20) containing 5% (w/v) non-fat milk, the membranes were incubated with specific primary antibodies against NLRP3, IL-1β, caspase-1, ASC, mTOR, p-mTOR, Akt, p-Akt, p-p38, p-JNK, p-ERK, or p-p65 at 4°C overnight in blocking solution, all antibodies were diluted at 1:1,000. Following three times of washed with TBST, the membranes were incubated with HRP-conjugated secondary antibodies at room temperature for 1 h. The chemiluminescence was detected using the ECL-chemiluminescent kit (Thermo Scientific) with Protein Simple (USA).

### Co-immunoprecipitation

Raw264.7 cells were lysed at 4°C in ice-cold cell lysis buffer and cell lysates were cleared by centrifugation (12,000 g, 10 min). Concentrations of proteins in the supernatant were determined by bicinchoninic acid (BCA) assay. Before immunoprecipitation, samples containing equal amounts of proteins were pre-cleared with various irrelevant IgG or specific antibodies (2–5 mg/ml) overnight at 4°C with gentle rotation and subsequently incubated with Protein A/G agarose/sepharose beads at 4°C with gentle rotation. Following 3 h incubation, agarose/sepharose beads were washed extensively with PBS for four times and proteins were eluted by boiling in 1×SDS sample buffer before SDS-PAGE electrophoresis.

### Measurement of mature IL-1β

Raw264.7 cells (1 × 10^6^) were cultured in six-well plates which infected with H37Ra for 4 h. Then washed twice with sterile PBS and replaced with 1 ml OPTI-MEM medium containing different concentration of baicalin (0, 12.5, 25, 50, or 100 μM). After 12 h, the supernatant of each well were concentrated according to the literature and with slightly modification (Shi et al., 2012). Removing 0.8 ml of the medium collected from each well and mixed with 0.8 ml methanol and 0.2 ml chloroform, vortexed, and centrifuged at 12,000 g for 5 min. The upper phase from each sample was removed and 0.8 ml methanol added. The samples were centrifuged again for 5 min at 12,000 g to remove the supernatant, and the pellet was placed in room temperature for 10 min to volatilize the methanol. Seventy milliliters of 1× loading buffer was added to each sample followed by boiling for 10 min prior to SDS-PAGE and immunoblotting with antibodies to detect mature IL-1β (AB-400-NA, R&D Systems). The adherent cells from each well were lysed with the above-mentioned lysis buffer and quantified before immunoblot to determine the cellular content of the different proteins.

### Immunofluorescence

Following the appropriate treatment, the cells were washed twice with PBS, fixed with 4% paraformaldehyde at room temperature for 10 min, and washed again with PBS. The cells were treated with penetrating reagents (0.2% of BSA, 2% of Triton X-100) for 10 min at 4°C and washed again with PBS, and then the cells were blocked with 5% bovine serum albumin for 30 min at room temperature. Rabbit anti-ASC, anti-LC3, anti-caspase-1, and anti-pp65 antibodies were used for immunofluorescence. Donkey anti-mouse IgG and goat anti-rabbit FITC conjugated antibodies were used as secondary antibodies. The nuclei were stained with DAPI at the concentration of 1 μg/ml for 10 min. In this experiment, confocal microscopy (LSM 880, Zeiss optics international trading co., LTD) was used for examination.

### Statistical analysis

Statistical analysis was performed by using SPSS 18.0 software (SPSS, Inc., Chicago, IL, USA). *P*-values were assessed by one-way analysis of variance (ANOVA), results were given as means ± standard deviations (SD). Data shown are representative of at least triplicate experiments. A value of *p* < 0.05 was considered to be statistically significant.

## Results

### Effect of baicalin on the viability of Raw264.7

**Figure 1:**
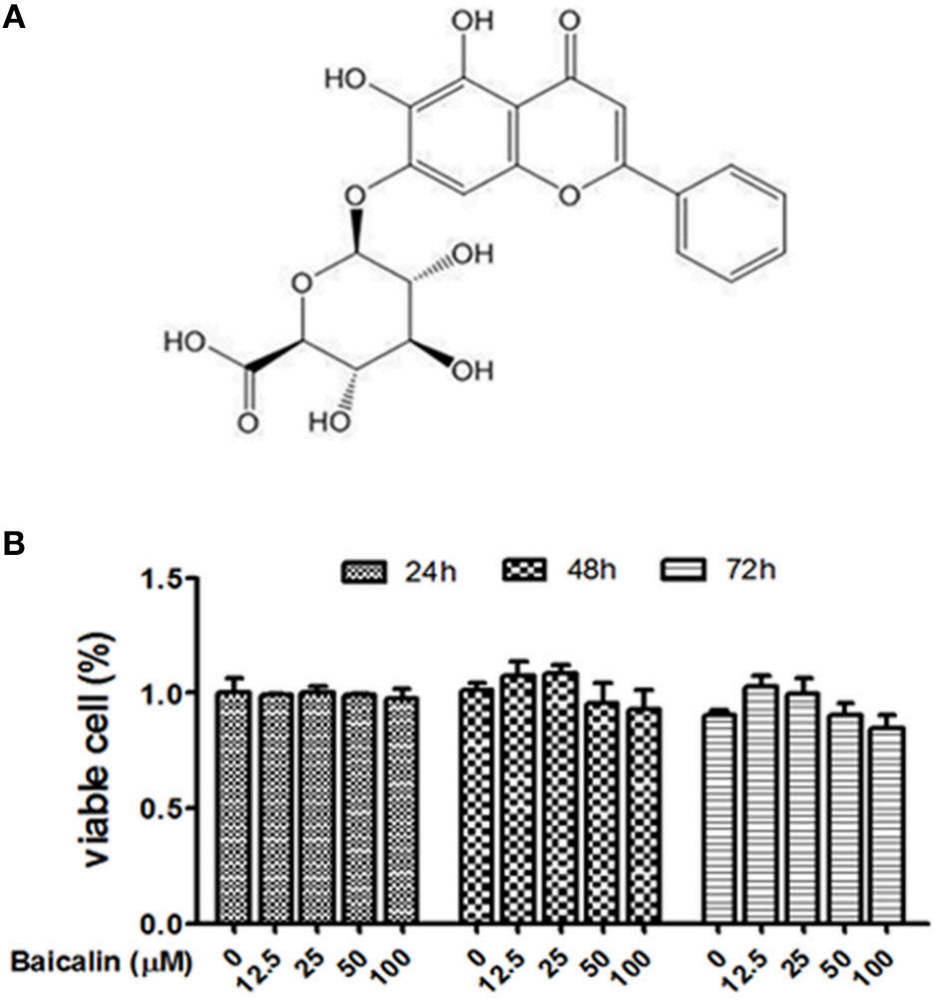
Effect of baicalin on the viability of Raw264.7. **(A)** The chemical structure of baicalin; **(B)** Proliferation assay was conducted to assess the cytotoxic effect of baicalin on Raw264.7. Data are shown as means ± SD of three independent experiments.

To optimize the concentration of baicalin, the cell viability assay was conducted to evaluate potential drug-induced toxicity. The proliferation of Raw264.7 cells was tested using the CCK-8 kit. As shown in Figure 1, baicalin (within 100 μM) did not affect the viability of Raw264.7 during observation periods (24, 48, or 72 h). Thus, the concentrations of baicalin within 100 μM were considered as safe for cells and could be used for the subsequent studies.

### Baicalin induces autophagy in Mtb infected macrophages

**Figure 2:**
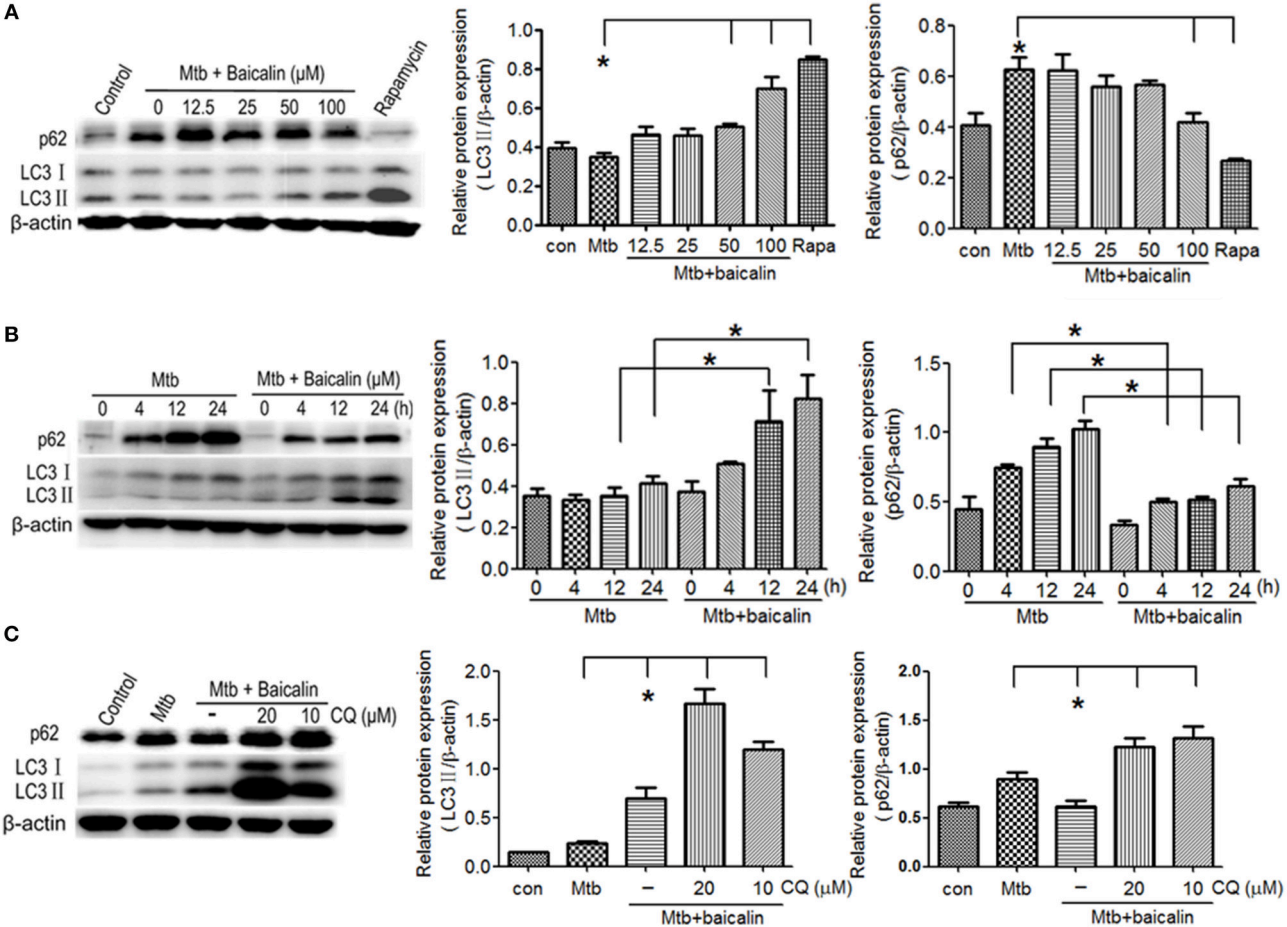
Baicalin can promote the activation of autophagy in Mtb-infected macrophages. **(A)** Western blot analysis of LC3 I/II and p62 expression in Mtb-infected Raw264.7 cells after treatment with different concentrations of baicalin (0, 12.5, 25, 50, 100 μM) or rapamycin (1 μg/ml) for 12 h. The right bar graphs show the statistical results for the relative quantitative expression of LC3 II and p62. **(B)** Western blot analysis of LC3 I/II and p62 expression in Mtb-infected Raw264.7 cells. After different times of Mtb infection (0, 4, 12, 24 h), cells were treatment with or without baicalin (100 μM). The right bar graphs showed the statistical results for the relative quantitative expression of LC3 II and p62. (**C)** Western blot analysis of LC3 I/II and p62 expression in Mtb-infected Raw264.7 cells after treatment with baicalin (100 μM) or CQ (10, 20 μM) for 12 h. The right bar graphs showed the statistical results for the relative quantitative expression of LC3 II and p62 expression. Data are shown with the means ± *SD* of at least three independent experiments. ^*^p < 0.05.

Mtb is an intracellular pathogen which can proliferate within infected macrophages by preventing the maturation of the phagosome where the bacteria reside. Autophagy represents a recognized cell-autonomous defense against intracellular pathogens that can employ various mechanisms for elimination of invasive Mtb (Gutierrez et al., 2004; Singh et al., 2006; Ponpuak et al., 2010). To assess the effect of baicalin on autophagy induction, we examined the expression of LC3 II and p62 (the biomarker of autophagosome) after treatment with different concentrations of baicalin (0, 12.5, 25, 50, or 100 μM). As shown in Figure 2A, baicalin induced the activation of autophagy in a dose-dependent manner and the concentration of 100 μM triggered most potent autophagy. The autophagy activation induced by baicalin (100 μM) was time-dependent (Figure 2B). To further validate the effect of baicalin on autophagy, we next evaluated the autophagic flux utilized with an autophagy inhibitor, chloroquine (CQ). As shown in Figure 2C, either 10 or 20 μM of CQ caused remarkable accumulation of LC3 and p62 compared to baicalin treatment. These data demonstrated that baicalin indeed induced the activation of autophagy in a concentration- and time-dependent manner.

### Baicalin has a significant killing effect on Mtb in macrophages

**Figure 3:**
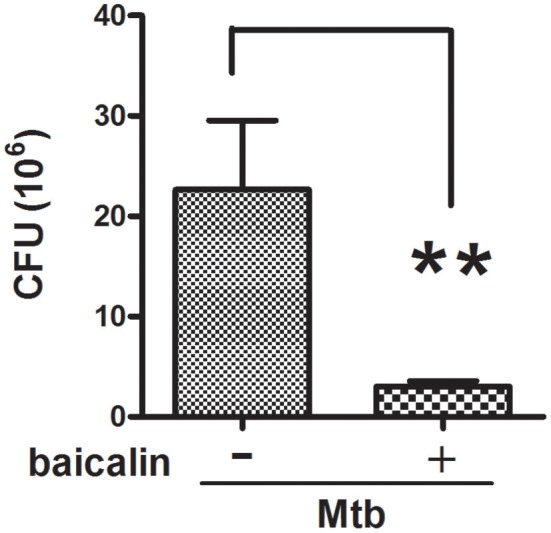
Inhibitory effect of baicalin on bacillary loads. Bacterial CFUs were counted after baicalin treatment for 48 h. ^**^p < 0.01, Data are shown as mean ± *SD* of three independent experiments.

Mtb, as a well-known intracellular pathogen, has developed several schemes (e.g., dampening the antimicrobial activity of ROS and RNS, preventing the maturation of early phagosomes, inhibiting the fusion of phagosome with lysosome, interrupting autophagy process) to escape from the antimicrobial mechanisms of macrophages and thus survive intracellularly (Awuh and Flo, 2017). Studies have shown that autophagy possesses the ability to eliminate intracellular bacteria. Since we have confirmed the induction effect of baicalin on autophagy (Figure 2), to assess the antibacterial effect of baicalin on Mtb, we performed the colony forming unit (CFU) counting assay. As shown in Figure 3, baicalin exerted an obvious antibacterial effect (the bactericide rate of baicalin reached 86.7% compared to untreated control) and this ability most likely relied on the induction of autophagy that further facilitated the killing of Mtb, as baicalin had no bactericidal effect on within 2.24 mM (data not shown).

### Baicalin suppresses the activation of NLRP3 inflammasome

**Figure 4:**
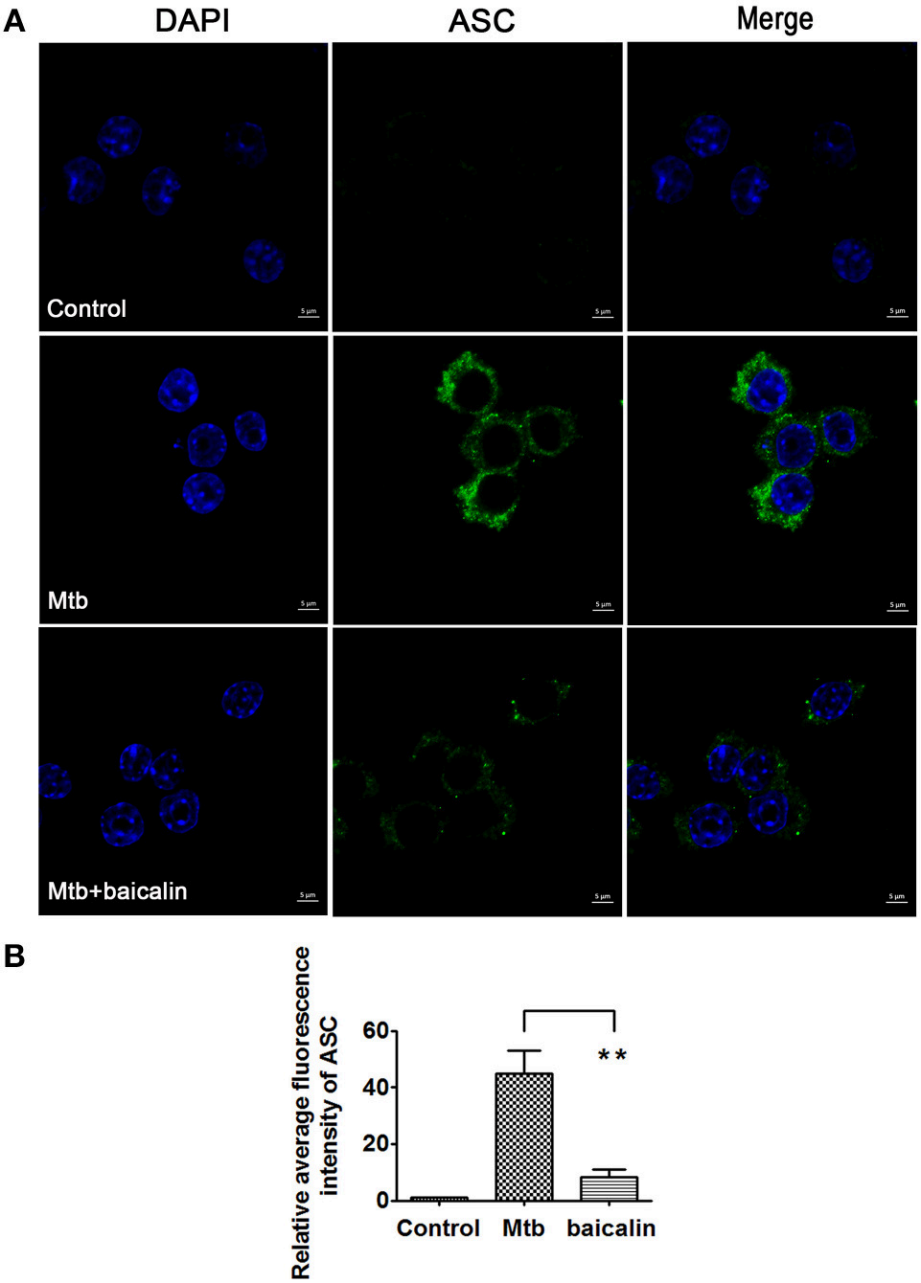
Baicalin inhibits the aggregation of ASC. **(A)** Immunofluorescence assay was performed and rabbit anti-ASC (green) and DAPI (blue) were used for immunostaining. Images were obtained with laser scanning confocal microscopy. **(B)** Quantification of the confocal images about ASC. The confocal images about ASC were quantified with ZEN 2011 Blue Version. Data are shown with means ± *SD*. ^*^p < 0.05.

**Figure 5:**
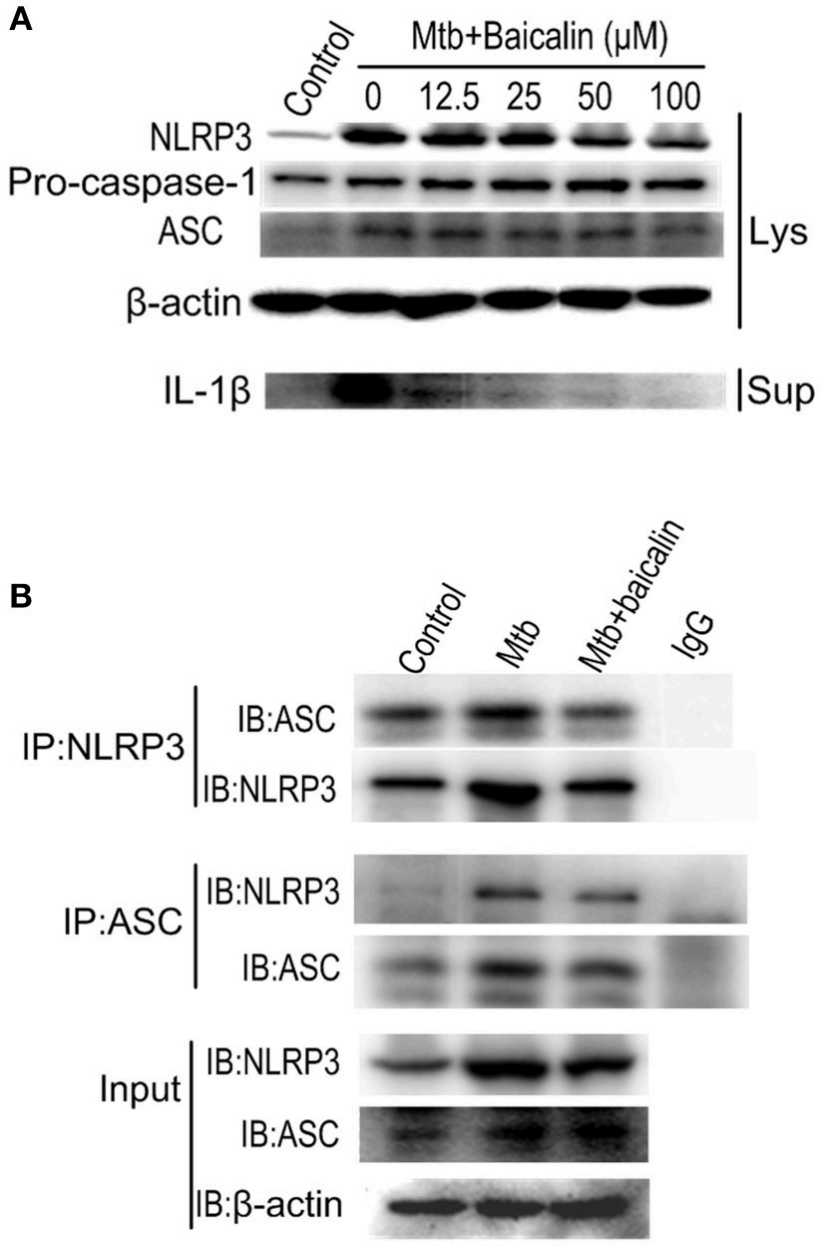
Baicalin evidently inhibits the Mtb-induced NLRP3 inflammasome activation. **(A)** Levels of NLRP3, ASC and pro-caspase-1 expression in cell lysates and the mature IL-1β in the supernatant were determined by immunoblot. **(B)** ASC or NLRP3 immunoprecipitates from Raw264.7 cells were immunoblotted for NLRP3 or ASC respectively, and re-blotted for ASC or NLRP3 respectively. Experiment performed three times.

**Figure 6:**
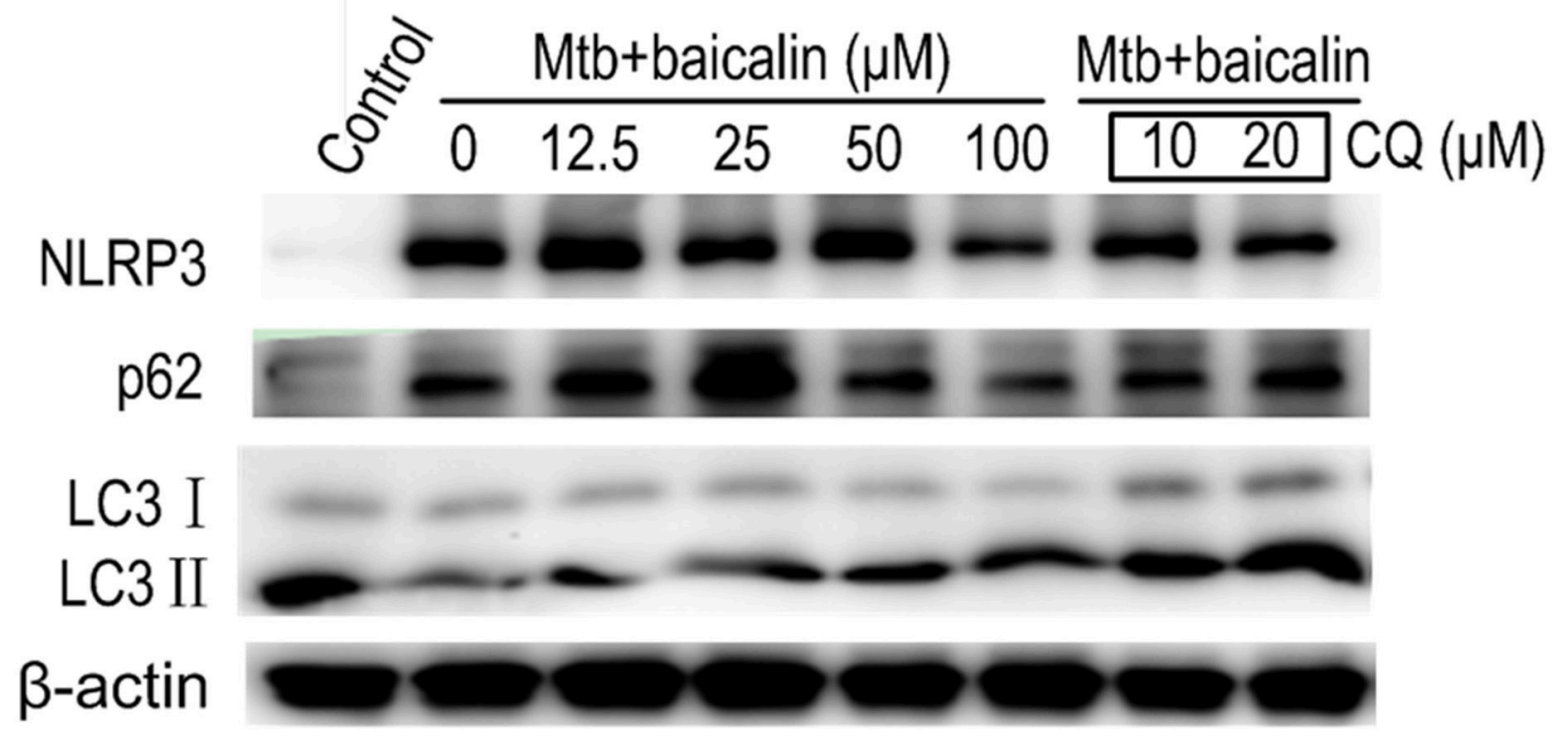
Baicalin inhibits the expression of Mtb-induced NLRP3 in primary peritoneal macrophage cells and chloroquine (CQ) prevented this effect. The expression of NLRP3, LC3 I/II and p62 in Mtb-infected primary peritoneal macrophage cells after treatment with different concentrations of baicalin or CQ (10, 20 μM) was detected by Western blot. The concentration of baicalin when used in combination with CQ was 100 μM.

Activation of inflammasome is an important post-transcriptional event to facilitate IL-1β release. Uncontrolled activation of inflammasome is associated with several inflammatory diseases, including TB (Wong and Jacobs, 2011; Mishra et al., 2013). NLRP3 inflammasome has been reported to contribute to the inflammatory tissue damage during mycobacterial infection (Kasahara et al., 1988; Mishra et al., 2013). Consistently, our immunofluorescence assay (Figure 4) indicated that Mtb triggered the accumulation of ASC which is essential for the activation of NLRP3 inflammasome or AIM2 inflammasome (Bryan et al., 2009; Mishra et al., 2010). We found that AIM2 was unchanged in Mtb-infected Raw264.7 regardless of the presence or absence of baicalin (data not shown). Moreover, Western blot showed that Mtb induced the increase of NLRP3, ASC, pro-caspase-1 which are the components of NLRP3 inflammasome (Figure 5A). On the contrary, baicalin treatment could either inhibit the formation of ASC specks in immunofluorescence (Figure 4) or decrease the NLRP3, ASC, pro-caspase-1 in a dose-dependent manner (Figure 5A). In addition, the inflammasome-derived IL-1β in the supernatant in Mtb infected macrophages was inhibited by baicalin. Furthermore, co-immunoprecipitation (Co-IP) showed that baicalin treatment restrained the interaction of NLRP3 with ASC (Figure 5B). Besides, in primary peritoneal macrophages, baicalin also showed inhibitory effect on the expression of NLRP3 and this inhibition ability was remarkably weakened when autophagy activity was blocked by CQ (Figure 6). ESAT-6 has been demonstrated the essential element to activate NLRP3 inflammasome (Mishra et al., 2010), although intracellular concentrations of ESAT-6 are similar in both H37Rv and H37Ra, the H37Ra remains defective for ESAT-6 secretion as the phoP gene mutation (Fortune et al., 2005). We speculate the potential mechanism on the activation of H37Ra-induced NLRP3 inflammasome that may be the case that *in vitro* cultures of the strain with some lysed bacteria may release ESAT-6 and therefore activate the inflammasome as observed in Figures 4–6. Together, our data indicated that baicalin could suppress the Mtb-induced NLRP3 inflammasome activation.

### Baicalin activates autophagy by inhibiting PI3K/Akt/mTOR signaling pathway instead of MAPK pathway

**Figure 7:**
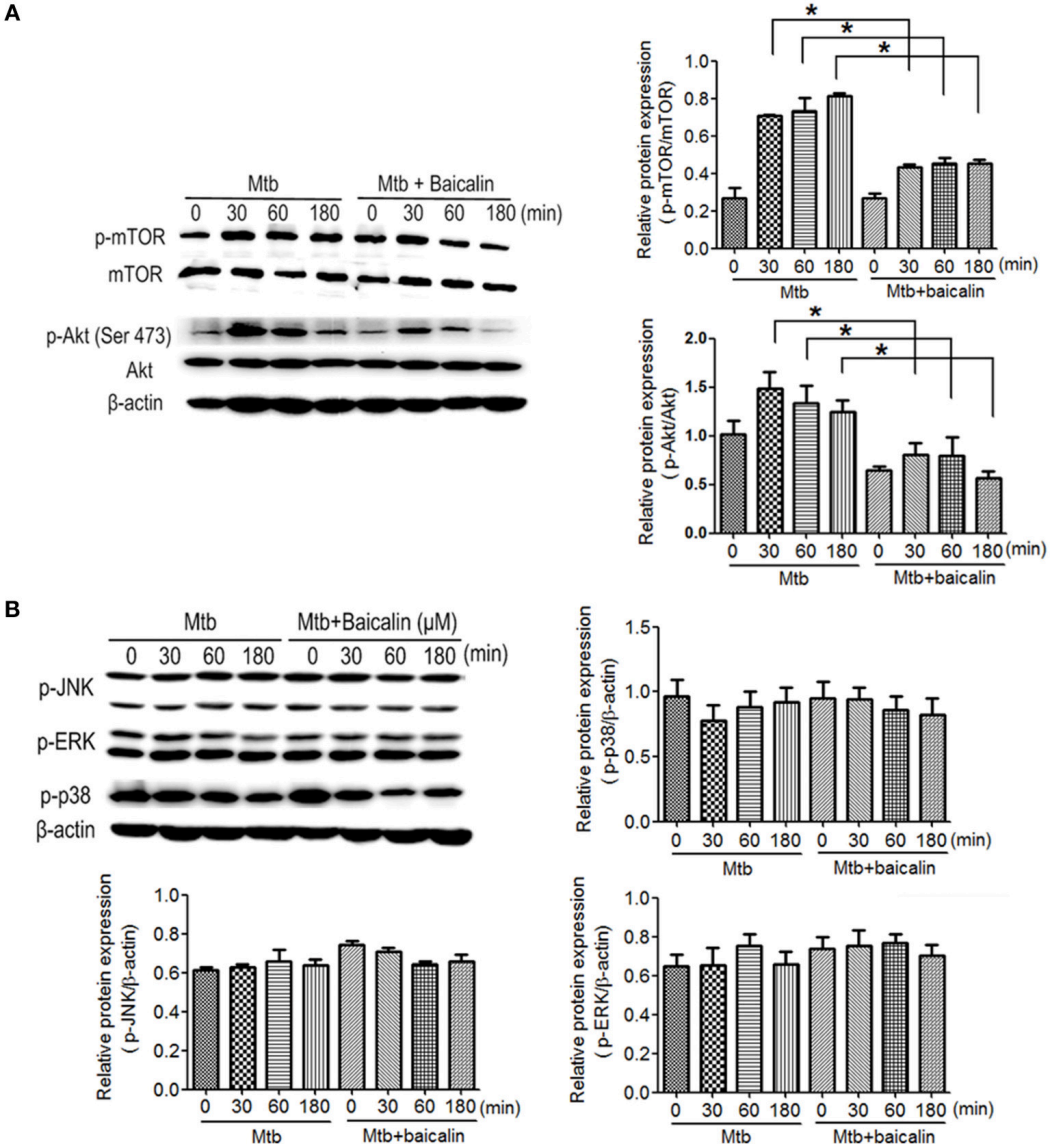
Bacalin suppresses the PI3K/Akt/mTOR pathway but has no effect on the MAPK signaling in Raw264.7 cells. **(A)** Western blot analysis of mTOR, p-mTOR, Akt, and p-Akt expression in Raw264.7 cells. β-actin was used as a control. The right bar graphs show the statistical results for the relative quantitative expression of p-Akt and p-mTOR. **(B)** Western blot analysis of p-JNK, p-ERK, and p-p38 expression in Raw264.7 cells. The right and below bar graphs show the statistical results for the relative quantitative expression of p-p38, p-ERK, and p-JNK. Data are shown with the means ± *SD* of at least three independent experiments. ^*^p < 0.05.

**Figure 8:**
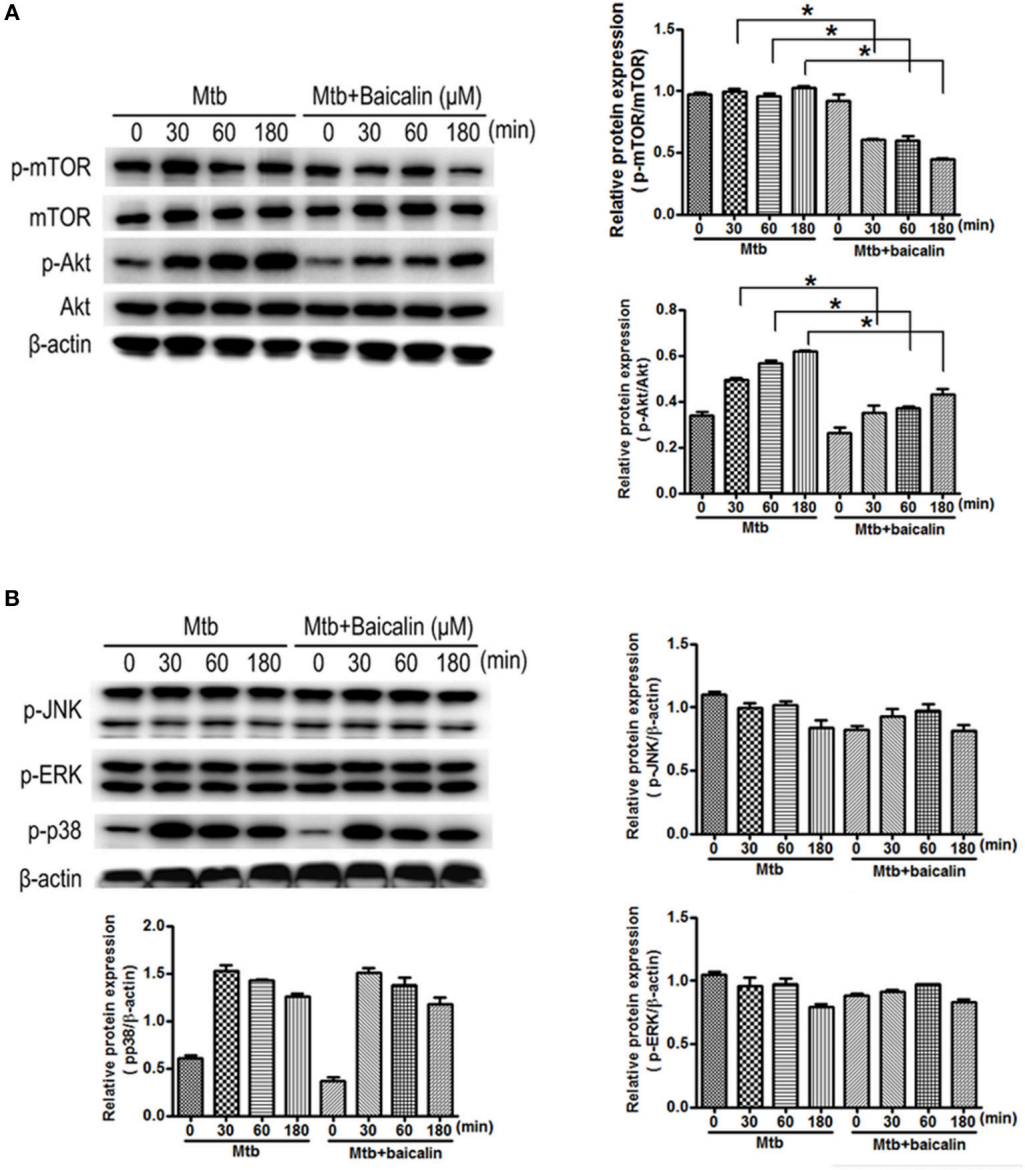
Bacalin suppresses the PI3K/Akt/mTOR pathway but has no effect on the MAPK signaling in primary peritoneal macrophage cells. **(A)** Western blot analysis of mTOR, p-mTOR, Akt, and p-Akt expression in primary peritoneal macrophage cells. β-actin was used as a control. The right bar graphs show the statistical results for the relative quantitative expression of p-Akt and p-mTOR. **(B)** Western blot analysis of p-JNK, p-ERK, and p-p38 expression in peritoneal macrophage cells. The right and below bar graphs show the statistical results for the relative quantitative expression of p-p38, p-ERK, and p-JNK. Data are shown with the means ± *SD* of at least three independent experiments. ^*^p < 0.05.

The activation of autophagy can be modulated by a wide range of signals. Inhibition of PI3K/Akt/mTOR and activation of MAPK pathway are known to induce autophagy activation. Then, we analyzed the effect of baicalin on these two signaling pathways. Results indicated that baicalin treatment inhibited the phosphorylation of Akt (Ser473) and mTOR (Ser2448) in a time-dependent manner both in Raw264.7 (Figure 7A) and peritoneal macrophages (Figure 8A). In addition, we also assessed the MAPK pathway and our results showed that baicalin had no effect on the phosphorylation of p38, JNK or ERK (Figures 7B, 8B). This is different with the reports that H37Rv could cause the activation of MAPK signaling pathway triggering secretion of inflammatory cytokines (Fietta et al., 2002; Jung et al., 2006). This difference is probably caused by the differences between the H37Ra and H37Rv strains (Jena et al., 2013). Although the H37Ra we used here is an attenuated strain which may cause lower toxic inflammatory reaction to host, but in terms of basic mechanism research, it is an irreplaceable good model for the study of intracellular TB as reported (Hart and Armstrong, 1974). Thus, our data revealed that baicalin inhibited the PI3K/Akt/mTOR signaling pathway instead of MAPK pathway to activate autophagy.

### Baicalin inhibits Mtb-induced NF-κB activation

**Figure 9:**
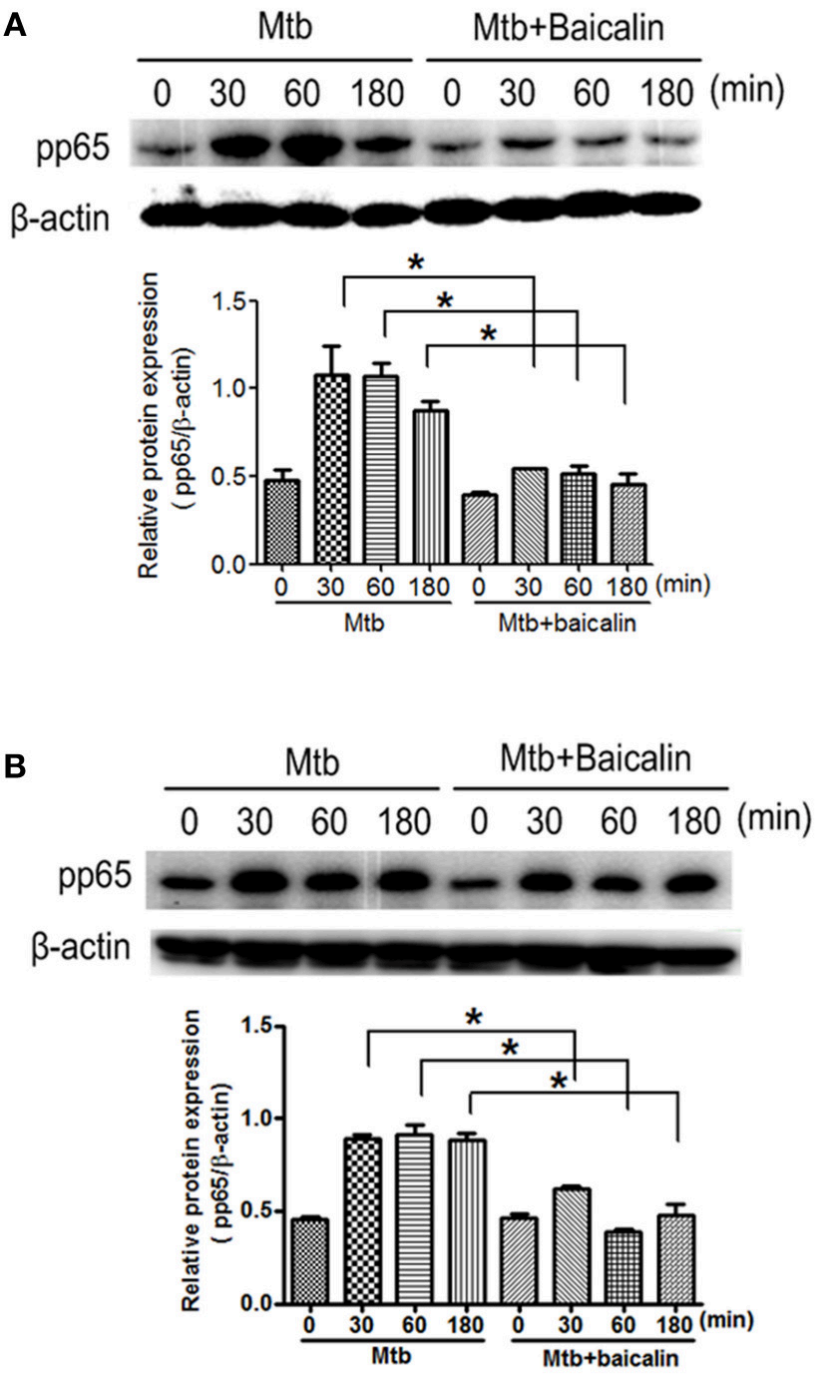
Baicalin inhibits Mtb-induced NF-κB activation. **(A)** Western blot analysis of p-p65 expression in Raw264.7 cells. β-actin was used as a loading control. The below bar graph shows the statistical result for the relative quantitative expression of p-p65. **(B)** Western blot analysis of p-p65 expression in primary peritoneal macrophage cells. β-actin was used as a loading control. The below bar graph shows the statistical result for the relative quantitative expression of p-p65. Data are shown as the means ± *SD* of at least three independent experiments. ^*^p < 0.05.

**Figure 10:**
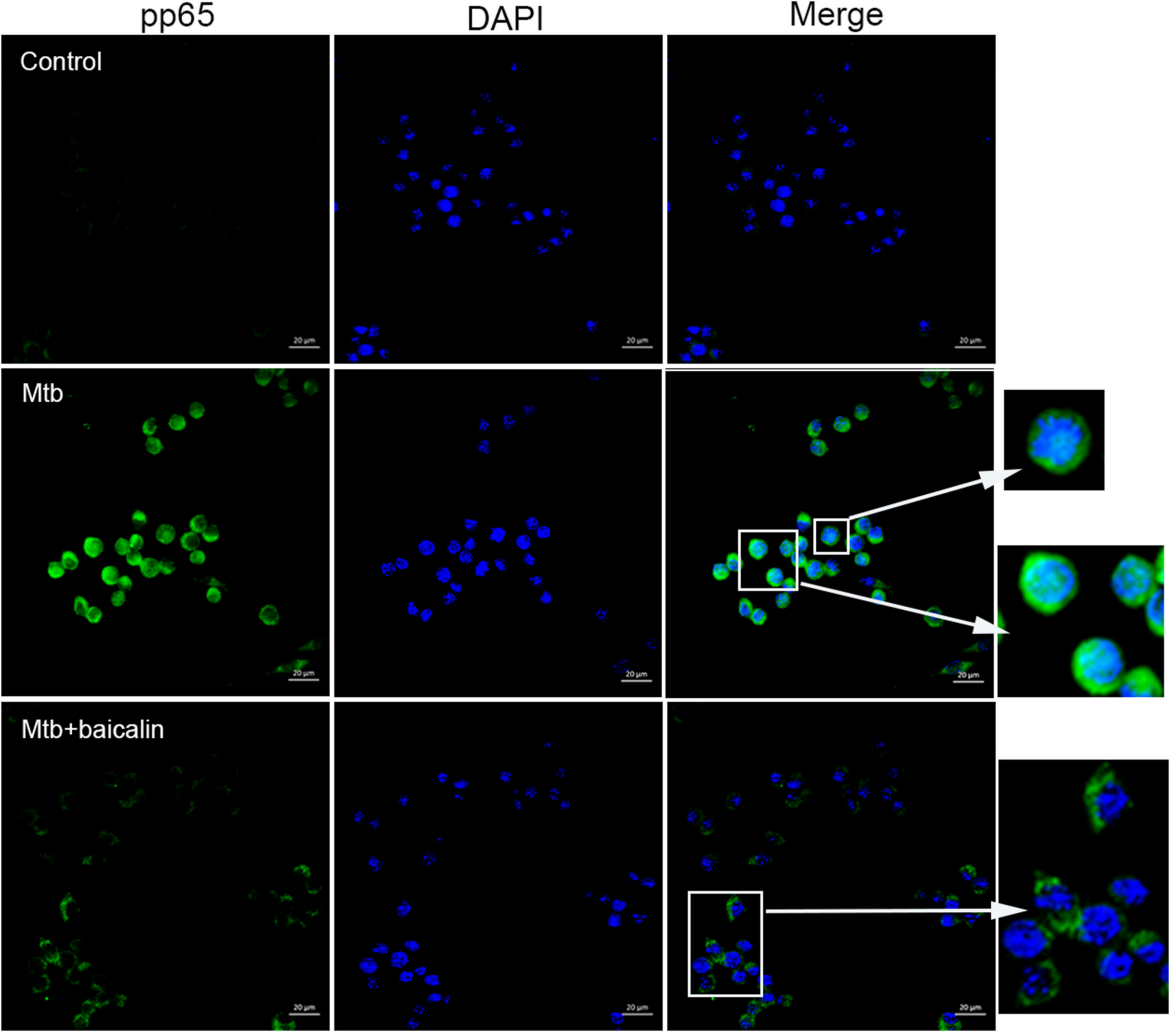
Baicalin prevents the nuclear translocation of Mtb-induced NF-κB. Confocal microscopy of Raw264.7 cells with different treatments immunostained with anti-pp65 (green) and DAPI (blue). Scale bars shown are 20 μm.

NF-κB plays essential roles in the activation of NLRP3 inflammasome (Ghonime et al., 2014; Lamkanfi and Dixit, 2014; He et al., 2016) and the transcriptional induction of various genes involved in inflammation. As shown in Figure 9, Mtb infection caused the activation of NF-κB signaling as evidenced by the elevated p65 phosphorylation levels both in Raw264.7 (Figure 9A) and primary peritoneal macrophages (Figure 9B). Confocal images indicated that Mtb increased nuclear entry of phosphorylated p65, while baicalin treatment markedly reduced the production of phosphorylated protein and prevented it from entering into the nucleus (Figure 10).

### Baicalin can induce co-localization of autophagosome and inflammasome

**Figure 11:**
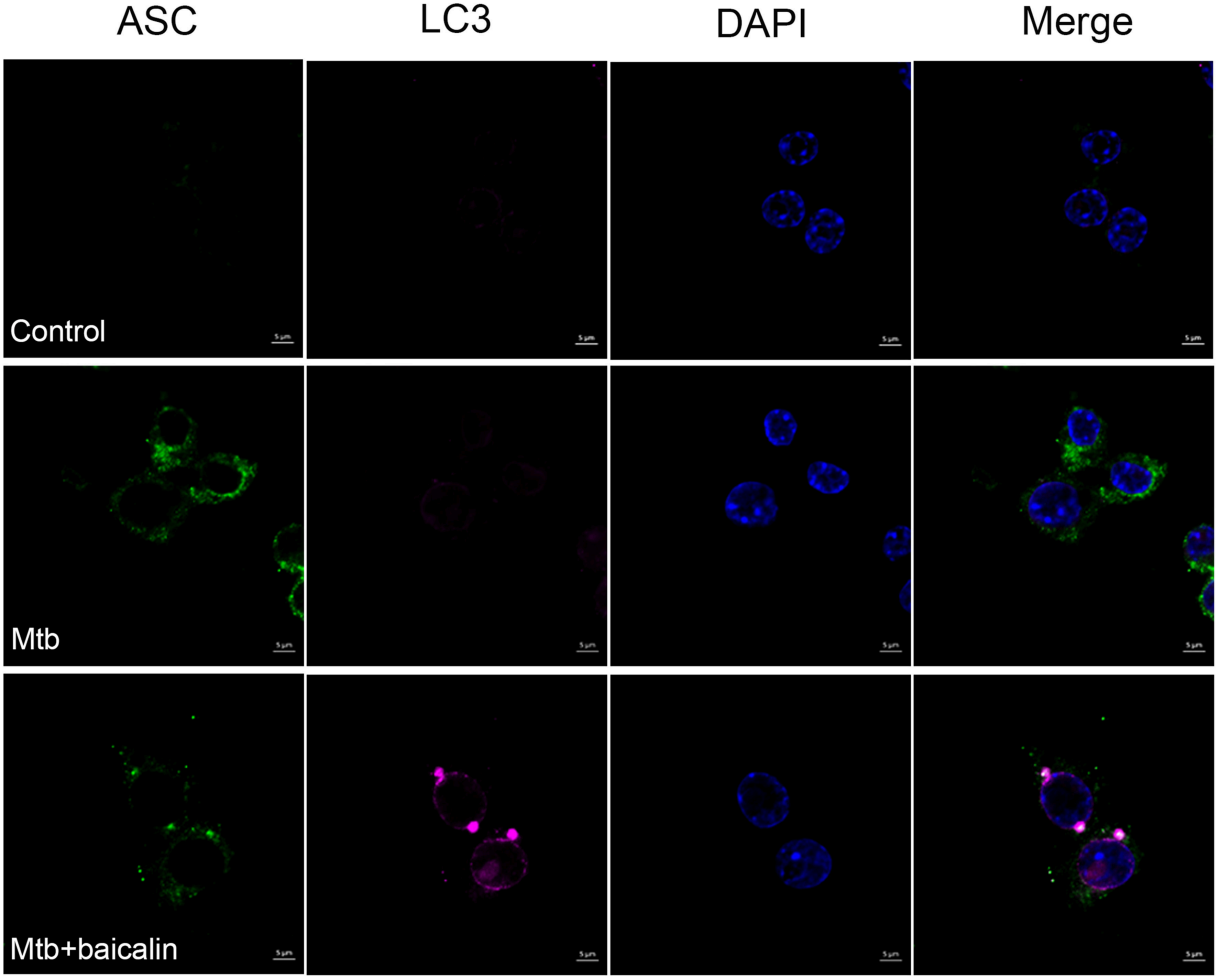
Baicalin promotes co-localization of inflammasome with autophagosome. Confocal microscopy of Raw264.7 cells with different treatments immunostained with anti-LC3 antibody (pink), anti-ASC antibody (green), and DAPI (blue). Scale bars shown are 5 μm.

Autophagy has been shown to play an important role in regulating inflammasome activation through the removal of inflammasome-activating endogenous signals or the sequestration and degradation of inflammasome components (Harris et al., 2011; Shi et al., 2012). We evaluated the relationship between them by means of immunofluorescence using confocal laser scanning microscope. As shown in Figure 11, Mtb induced the accumulation of ASC protein which represented the activation of NLRP3 inflammasome, and baicalin treatment decreased the ASC specks. Meanwhile, baicalin elevated the production of LC3 and induced the co-localization of LC3 and ASC, which is representative of autophagosome and inflammasome respectively. Thus, we conclude that baicalin induced autophagy activation, inhibited the activity of NLRP3 inflammasome through autophagic degradative mechanism, and restrained the secretion of IL-1β.

## Discussion

**Figure 12:**
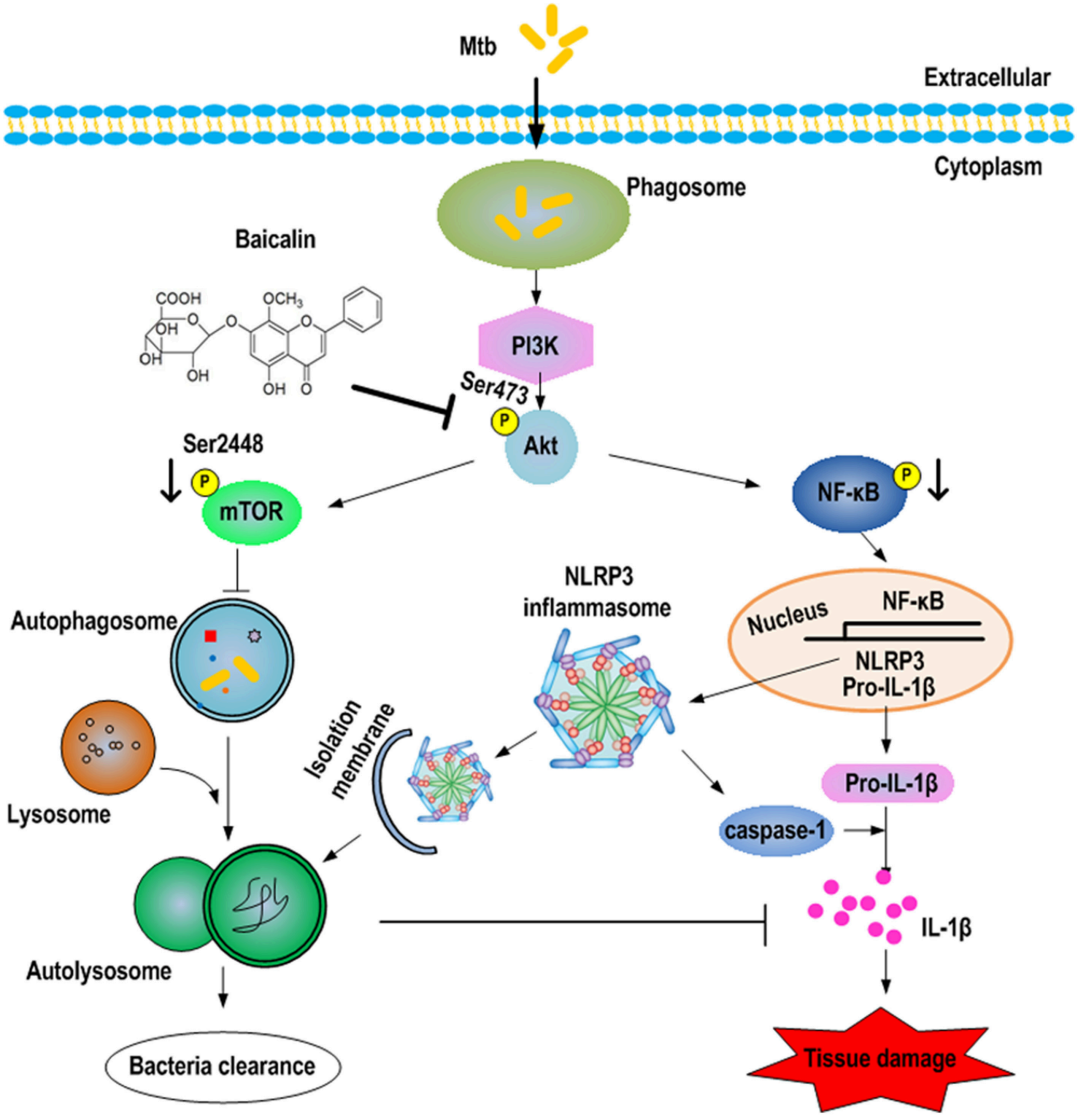
Schematic summary of baicalin-driven regulation of autophagy and inflammation during Mtb infection. Baicalin inhibits the phosphorylation of Akt, thus inducing the activation of autophagy through PI3K/Akt/mTOR signaling pathways leading to bactericidal and inflammasome-inhibitory effect. Furthermore, baicalin inhibits the PI3K/Akt/NF-κB pathway contributing to the inhibitory effect on NLRP3 inflammasome activation.

This study first reveals the roles of baicalin-mediated autophagy inducing effect in protection against Mtb infection. Our work demonstrated that baicalin inhibited the phosphorylation of Akt/mTOR thereby inducing autophagy to kill intracellular Mtb and showed suppressing effect on the activation of NLRP3 inflammasome and NF-κB signaling pathway triggered by Mtb (as shown in Figure 12). Baicalin is a safe, effective and widely available herb monomer which can be extracted from plants of genus Scutellaria (grown in Asian countries including China) or Oroxylum indicum (grown in other countries) (Dinda et al., 2017). The extracts from the roots of Scutellaria baicalensis are widely used in traditional Chinese medicines to treat various diseases such as hepatitis, atherosclerosis, dysentery, as well as common cold and other respiratory disorders (Li-Weber, 2009). Consistent with our work, previous studies have reported that baicalin was capable of inducing autophagy to cause cancer cell autophagic death (Zhang et al., 2012; Lin et al., 2013). Unlike the results of these publications and our research, there a study reported that baicalin inhibited influenza A virus induced autophagy (Zhu et al., 2015). As for TB, to our knowledge, this is the first demonstration that baicalin has antimycobacterial activity via induction of autophagy of host macrophages.

Induction of autophagy in Mtb-infected macrophages by several means (physiologically, immunologically, or pharmacologically) has been shown to kill Mtb (Gutierrez et al., 2004). Subsequent studies have extended the initial observation and established the key role of autophagy in defense against TB (Deretic, 2008; Harris et al., 2009; Jo, 2013). Consistent with these data, our results demonstrated that baicalin stimulated the activation of autophagy as evidenced by the upregulation of LC3II and downregulation of p62 and the unobstructed autophagic flux process after baicalin treatment. In addition, baicalin caused inhibition of intracellular Mtb replication. We attributed this killing effect to baicalin-stimulated autophagy effect because baicalin showed no direct impact on Mtb proliferation at 300 μM in our *in vitro* study. Autophagy has anti-inflammatory activity and protects the host from tissue necrosis and lung pathology (Castillo et al., 2012). The existing reports agree that autophagy negatively regulating inflammasome activation through a variety of mechanisms (Saitoh et al., 2008; Harris et al., 2011; Nakahira et al., 2011; Zhou et al., 2011; Shi et al., 2012; Lupfer et al., 2013; Martins et al., 2015; Saitoh and Akira, 2016). Inflammasome has been recognized to be involved in the pathological progress of Mtb infection (Carlsson et al., 2010; Wong and Jacobs, 2011; Castillo et al., 2012; Dorhoi et al., 2012; Mishra et al., 2013) causing granulomatous lung lesions and systemic inflammatory responses (Bekker et al., 2000). Although granulomas have long been considered to benefit the host by containing and restricting mycobacteria, recent studies have demonstrated that tuberculous granuloma provides a safety shelter for bacterial growth, persistence, and proliferation (Davis and Ramakrishnan, 2009; Silva Miranda et al., 2012; Cambier et al., 2014) and even caused lung damage (Bekker et al., 2000; Philips and Ernst, 2012). Our data indicated that baicalin inhibited the Mtb-induced inflammation process by restraining the NLRP3 inflammasome activation and subsequent production of inflammatory mediators triggered by Mtb infection. Complementing anti-TB drugs with anti-inflammation interventions could improve treatment efficiency and outcome evidenced by reports that patients treated with corticosteroids in conjunction with TB drugs contribute to a modest decrease in mortality and is helpful in extrapulmonary tuberculosis including meningitis and pleural disease (Critchley et al., 2013; Prasad et al., 2016). Nevertheless, considering the strong immunosuppressive effects and many other side effects, caution is needed when applying corticosteroids in pulmonary TB. In contrast, baicalin possess the advantage over corticosteroids that has no side effects such as immunosuppression. Moreover, baicalin has been demonstrated to inhibit sterile (Wang et al., 2016) or bacterial inflammation (Guo et al., 2013; Liu et al., 2017) and showed immunoprotective effect in the sepsis model (Hu et al., 2015). Hence, baicalin could be a new candidate for the development of adjunctive anti-TB therapies.

Having confirmed the antimycobacterial and anti-inflammatory effects of baicalin, we next explored the functional mechanisms. Two classical autophagy related signaling pathways were assessed, the PI3K/Akt/mTOR signaling pathway which has been recognized as negatively regulating the activation of autophagy (Heras-Sandoval et al., 2014), and the MAPK pathway which acts as a positive regulator of autophagy (Krishna and Narang, 2008; Zhou et al., 2015). Data indicated that baicalin inhibited the phosphorylated Akt (Ser473) and mTOR (Ser2448) but without influencing phosphorylated JNK, ERK, or p38. Previous studies have suggested that baicalin showed evident inhibitory effect on NF-κB activation (Guo et al., 2013; Fu et al., 2016). Here, our data showed that baicalin significantly inhibited the phosphorylation of NF-κB and prevented its entry to the nucleus. Based on our findings, we conclude that baicalin actually targeted PI3K/Akt to restrain the NF-κB activity, which is consistent with previous studies (Kang et al., 2012; Guo et al., 2016).

Taken together, our data demonstrated that baicalin has both anti-inflammatory and antimycobacterial effects on Mtb-infected macrophages and this endows baicalin the great potential as a candidate of HDT for new anti-TB adjuvant therapy. Additionally, better understanding the basic biology of mycobacterial pathogenesis may guide the search for more effective and specific HDT targets. Drugs like baicalin that manipulate host cellular defense mechanisms such as autophagy could achieve bactericidal and anti-inflammatory effects may provide new opportunities to combat intracellular pathogens like Mtb. Future studies are needed to evaluate baicalin in conjunction with TB drugs for improved treatment of TB in animal models and if promising in humans.

## Author contributions

XJ and YiZ conceived and designed the experiments. QZ, JS, YW, WH, and JW performed the experiments. XJ, YiZ, QZ, JS, YuZ, and LW analyzed the data. QZ, JS, XJ, and YiZ wrote the paper. All authors have read and approved the final manuscript.

### Conflict of interest statement

The authors declare that the research was conducted in the absence of any commercial or financial relationships that could be construed as a potential conflict of interest.

## Abbreviations

TB: tuberculosis
Mtb: *Mycobacterium tuberculosis*
WHO: World Health Organization
HDT: Host-directed therapy
ASC: apoptosis-associated speck-like protein containing a caspase recruitment domain
PI3K: phosphatidylinositol 3 kinase
Akt: protein kinase B
mTOR: mammalian target of rapamycin
MAPK: mitogen activated protein kinases
NF-κB: nuclear factor-kappa B
NLR: NOD-like receptor
DMEM: Dulbecco's Modified Eagle's Medium
CFU: Colony forming unit
BCA: bicinchoninic acid
PBS: phosphate-buffered saline
BSA: bovine serum albumin
CQ: chloroquine
Con: control
Lys: lysate
Sup: supernatant
rapa: rapamycin.
